# Subacute thyroiditis and different SARS-CoV-2 vaccine

**DOI:** 10.20945/2359-3997000000570

**Published:** 2022-11-28

**Authors:** Rujittika Mungmunpuntipantip, Viroj Wiwanitkit

**Affiliations:** 1 Bangkok Thailand Private Academic Consultant, Bangkok, Thailand; 2 Dr DY Patil University Pune India Honorary professor, Dr DY Patil University, Pune, India

**DEAR EDITOR**

We would like to share ideas regarding the article “Two cases of subacute thyroiditis (SAT) after different types of SARS-CoV-2 vaccination” (1). Bostan and cols. mentioned that they “described two cases who developed SAT three days after the messenger RNA vaccine against COVID-19 (Pfizer-BioNTech^®^) … (CoronaVac^®^) (1)” and concluded that “physicians should be aware of SAT that may occur within a few days following the COVID-19 vaccination” (1).

The effect of the COVID-19 vaccination on endocrine system is an interesting issue. The thyroid problem after COVID-19 vaccination is sporadically reported and it is an important consideration in clinical endocrinology. The abnormal thyroid profile after vaccination is possible and there are several reasons. The immunopathology triggered by vaccination might be a possible underlying pathogenesis. However, it is necessary to rule out the pre-vaccination subclinical thyroid disorder and other concurrent medical issues that might result in thyroid problem (2). Finally, the abnormal thyroid function after vaccination might be a result of hyperviscosity induced by COVID-19 vaccination (3,4). Those mentioned possibilities should be recognized and carefully investigated in any SAT cases firstly detected in COVID-19 vaccine recipient.
